# De Novo Design of a Highly Stable Ovoid TIM Barrel: Unlocking Pocket Shape towards Functional Design

**DOI:** 10.34133/2022/9842315

**Published:** 2022-10-10

**Authors:** Alexander E. Chu, Daniel Fernandez, Jingjia Liu, Raphael R. Eguchi, Po-Ssu Huang

**Affiliations:** ^1^Biophysics Program, Stanford University, Stanford, CA, USA; ^2^Department of Bioengineering, Stanford University, Stanford, CA, USA; ^3^Program in Chemistry, Engineering, And Medicine for Human Health (ChEM-H), Stanford University, Stanford, CA, USA; ^4^Stanford ChEM-H, Macromolecular Structure Knowledge Center, Stanford University, Stanford, CA, USA; ^5^Department of Biochemistry, Stanford University, Stanford, CA, USA; ^6^Bio-X Institute, Stanford University, Stanford, CA, USA

## Abstract

The ability to finely control the structure of protein folds is an important prerequisite to functional protein design. The TIM barrel fold is an important target for these efforts as it is highly enriched for diverse functions in nature. Although a TIM barrel protein has been designed de novo, the ability to finely alter the curvature of the central beta barrel and the overall architecture of the fold remains elusive, limiting its utility for functional design. Here, we report the de novo design of a TIM barrel with ovoid (twofold) symmetry, drawing inspiration from natural beta and TIM barrels with ovoid curvature. We use an autoregressive backbone sampling strategy to implement our hypothesis for elongated barrel curvature, followed by an iterative enrichment sequence design protocol to obtain sequences which yield a high proportion of successfully folding designs. Designed sequences are highly stable and fold to the designed barrel curvature as determined by a 2.1 Å resolution crystal structure. The designs show robustness to drastic mutations, retaining high melting temperatures even when multiple charged residues are buried in the hydrophobic core or when the hydrophobic core is ablated to alanine. As a scaffold with a greater capacity for hosting diverse hydrogen bonding networks and installation of binding pockets or active sites, the ovoid TIM barrel represents a major step towards the de novo design of functional TIM barrels.

## 1. Introduction

Significant advances in computational methods have enabled the de novo design of proteins of diverse folds and topologies, including all-*α*, all-*β*, and mixed *α*-*β* folds [1, 2]. Due to their simple structural features and high thermodynamic stability, these de novo proteins have been used to study determinants of protein folding and stability, develop specific protein-protein interaction systems, generate self-assembling protein nanoparticles, design protein and small molecule ligand binders, stabilize scaffolds for epitope presentation, and more [3–8]. At the core of these remarkable achievements is the ability to control elements of protein structure and an understanding of the relationship between sequence, structure, and stability.

The TIM barrel (TIMB) fold is a critical target of these efforts due to its ubiquity in nature across highly diverse functional families, suggesting its broad utility as a design scaffold [9–11]. In particular, its amenability to functional design, as evidenced by its prevalence among natural enzymes (nearly 10 percent are TIMBs) and its enrichment in computational protocols for constructing de novo active sites, may be in part due to its simple structural organization of repeating *β*/alpha units connected by variable loops [10, 12–14]. Exploiting this organization, Huang et al. designed a de novo TIMB protein, sTIM11, by employing basic structural principles of the fold and rigorously examining viable syntaxes and sequences [15]. Here, we use the word “syntax” to refer to the topological organization of a protein, augmented with information about the specific lengths of each secondary structure element. This work provided both a stable, idealized protein scaffold which could be more easily redesigned for various functional purposes, as well as a deeper understanding of the structure and sequence determinants of the TIMB fold. Subsequent work has validated the design of the de novo TIMB, investigating and improving its stability, adding additional structural elements found in natural TIMBs, and installing a lid domain to facilitate metal binding [16–18].

However, exploring beyond the principles learned in the design of the original de novo TIMB is important for generating structural variants better equipped for installing function. Several features inherent to the design of the de novo TIMB limit its capacity for supporting functional elements. In natural TIMBs, the *β*-*α* loops play an important role in ligand recognition and catalytic residue positioning and exhibit a broad spectrum of lengths and conformations [19, 20]; in the de novo TIMB, half of these loops are designed to be “structural.” Four of the eight loops participate in key hydrogen bonding interactions that maintain the geometric arrangements from one repeat unit to the next, which limits both the variability accessible by the loops and also the space needed to form a significant hydrophobic core for improved stability. More fundamentally, the *β* barrel plays a key role in the positioning of active site residues in a way that is not simply rectified by altering loop conformations, and the ability to control and vary *β* barrel curvature allows for insertion of new, more complex residue combinations and networks that may not be possible in a circular TIMB. Natural TIMBs exhibit great diversity in this element, ranging from “circular” TIMBs to elongated or “ovoid” TIMBs, as well as other nonsymmetrical arrangements in between. Indeed, the eponymous TIMB enzyme, triosephosphate isomerase, exhibits an ovoid *β* barrel curvature. In sTIM11 and all subsequent variants, the *β* barrel is circular and has limited structural designability due to its central relationship to the fold architecture, determining in part both the lengths and orientations of *α* helices and connecting loops. This problem is exacerbated by the fact that TIMB stability is heavily dependent on hydrophobic packing and charge interactions in the *β* barrel [21, 22]. Both the presence of structural loops and the rigid curvature of the *β* barrel have made it challenging to create functional variants of the de novo TIMB.

Thus far, while additions and modifications have been made to the original de novo TIMB [17, 18], attempts to coerce the *β* barrel to adapt new geometry through mutations and sequence redesign have remained futile, suggesting that the principles deduced from the circular TIMB design may not be generalizable to alternative geometries. This is despite significant progress in *β* barrel design, including an idealized syntax for the TIMB fold and successful “single-wall” de novo *β* barrel proteins [4, 15, 23]. In particular, it is difficult to directly reuse strategies for controlling single-wall *β* barrel shape in TIMB design. Because the *β* strands play a smaller role in determining TIMB architecture (due to the alternating *β*/*α* arrangement and the relatively short strands in a TIMB *β* barrel), strategies that induce control over *β* barrel shape, such as *β*-bulges and glycine corners, are difficult to effectively transfer from single-wall *β* barrels to TIMBs. Similarly, the fourfold symmetry of sTIM11 makes it difficult to induce symmetry breaking and ovoid curvature using sequence mutations alone. Taken together, this indicates that defining a new twofold symmetric syntax via de novo design is necessary for control over TIMB *β* barrel curvature.

Here, we wanted to investigate whether the curvature and eccentricity of the central *β* barrel could be altered through de novo design while also eliminating structural loops and improving stability. To this end, we report the computational design and experimental validation of a de novo ovoid TIMB protein, with a completely novel syntax and sequence. The designed protein exhibits an elongated *β* barrel architecture with loops which are not structurally involved and a more developed hydrophobic core. In accordance with our design goals, the protein folds solubly and is highly stable. The crystal structure is in close agreement with the design model, demonstrating the effectiveness of our design hypotheses. The extended shape of the ovoid TIMB allows the installation of more diverse residue identities and combinations, facilitating several tests of stability. The interior of the barrel is highly robust to mutation, supporting buried polar and charged residues and even ablation to polyalanine. Our de novo designed ovoid TIMB represents a highly improved version of the original TIMB with many more potential avenues for downstream functionalization.

## 2. Materials and Methods

### 2.1. De Novo Computational Design

RosettaRemodel [24] was used to sample secondary structure arrangements from a collection of protein structure fragments to generate a protein backbone and then to design the side chain sequence conditioned on sampled backbone. To limit the search complexity of any individual sampling run, we designed OT1 with an autoregressive procedure. Beginning with the initial *β*1-*α*1-*β*2-*α*2 unit, each successive secondary structure element was sampled individually: the *β*3 unit was sampled with *β*1-*α*1-*β*2-*α*2 held constant, and a promising design was selected, then the *α*3 unit was sampled with *β*1-*α*1-*β*2-*α*2-*β*3 held constant, and a promising design was selected, and so on with *β*4. Promising designs were selected by manual inspection of conformity to structural ideality (*β* strands pairing, helices positioned correctly, loop backbone torsions in permitted Ramachandran space, loop backbone hydrogen bonds satisfied). To sample *α*4, we sampled the *α*4 helix jointly with the entire second half of the structure using the -repeat_structure option of the RosettaRemodel, conditioned on all previous elements. (Since OT1 was designed as a twofold repeat protein, the second half is identical to the first half.) In this step, promising designs were selected by first ranking all designs by *α*/*β* barrel probability under a fold-classification model (averaging over all residues) [25] and then manual inspection of high probability designs using the same ideality criteria as above.

With ideal backbones identified, de novo sequences were designed by interleaving rounds of side chain and rotamer sampling with gradient-based Rosetta energy minimization. First, automated design was conducted with the relax/minimization step constrained to starting coordinates, to prevent changes to the *β* barrel curvature. Once the backbone architecture was stabilized, we refined the sequence using three rounds of an “iterative enrichment” protocol [26]. Each residue position was classified by manual inspection as a hydrophobic, solvent-exposed, or boundary (could be hydrophobic or polar) position. Hydrophobic and boundary positions were designed first for several rounds, holding the polar positions constant. The distribution of amino acids for each position was calculated for ~1,000 Rosetta trajectories, and the sequence design space was restricted to those represented in the distribution for each position. After these positions were fairly converged, the solvent-exposed positions were designed in a similar manner, until convergence. Finally, each position in the final, lowest-energy designs was manually curated, and erroneous or pathological designs were removed. During each stage of sequence design, distance constraints between atoms involved in backbone hydrogen bonding of the *β* barrel were enforced to maintain structural fidelity. Example Rosetta flags/options and Remodel blueprints for sequence design are provided in Fig. S1. To conduct *ab initio* structure prediction, for each initial design, 3-mer and 9-mer fragments were picked with Rosetta and 25,000 *ab initio* prediction trajectories were initiated and run with the AbInitioRelax protocol [27].

### 2.2. Protein Expression and Purification

Genes encoding the designed sequences and a N-terminal 6xHis tag were ordered from Integrated DNA Technologies and Twist Biosciences and cloned into the pET24a expression vector using Gibson assembly. Resulting plasmids were sequenced to confirm fidelity to the design and transformed into the E. coli BL21 (DE3) strain. Single colonies were picked and used to inoculate 5 mL starter cultures, which were grown to saturation and used to inoculate 0.5 L or 1 L main cultures. Both starter and main cultures were grown using 2xYT media (Research Products International) with 30 mg/L final concentration kanamycin. Main cultures were induced in late log phase with 0.5 mM final concentration IPTG, and the protein was expressed overnight at 16°C.

The cells were pelleted and resuspended in 30 mL of 50 mM Tris, pH 7.5, and frozen at -80°C and then thawed and lysed at room temperature with 0.6 mL 600 mg/mL lysozyme (10.17 mg/mL final concentration), 0.6 mL 50 mM PMSF (0.85 mM final concentration) (Roche), and 4.2 mL 4 M NaCl (474.58 mM final concentration), followed by sonication or microfluidization on ice. Cell debris was then removed by centrifugation at 20,000 *g* for 30 min. The supernatant was separated from the pellet and loaded on to Ni-NTA beads (Thermo Scientific) in gravity columns at room temperatures, washed with phosphate-buffered saline (PBS; Santa Cruz Biotechnology) with 50 mM imidazole, and eluted with stepwise 1 mL fractions of PBS with 0.5 M imidazole. Protein expression was assessed by SDS-PAGE and Coomassie SafeBlue staining (Thermo Scientific) or absorbance on a gel reader (Bio-Rad). Fractions containing protein of interest were then pooled and concentrated on Amicon Ultra-4 10000 Da MWCO centrifugal filters. This was followed by fast protein liquid chromatography on an AKTA Purifier system (GE Life Sciences) using a 1 mL HiTrap Q HP anion exchange column (GE Life Sciences) and/or a Superdex 75 10/300 GL size exclusion gel filtration column (GE Life Sciences). All proteins underwent immobilized metal affinity chromatography (IMAC) and size exclusion chromatography (SEC); only protein preparations which were used for crystallography underwent anion exchange, which was after IMAC and before SEC. For anion exchange, the sample was injected in 0.5× PBS with 250 mM imidazole and eluted in a linear gradient from 50 mM to 800 mM NaCl over 15 column volumes.

### 2.3. Protein Secondary Structure and Stability with Circular Dichroism

Circular dichroism (CD) spectra were collected on a Jasco J-815 CD spectrometer. Protein in PBS solution (Santa Cruz Biotechnology) was diluted to approximately 0.2 mg/mL and placed in a cuvette with a pathlength of 1 mm. The CD signal was measured at 20°C at each unit wavelength from 200 to 260 nm at 1 nm/s and a D.I.T. of 1 s. A spectrum with a blank sample of pure buffer was taken before taking the protein spectra to measure the background signal; if significant background was measured, the cuvette was cleaned by heating and washing with Contrad-10 detergent. For thermal denaturation, the sample was then heated from 20°C to 95°C at a rate of 1°C/min, measuring the CD signal at each degree change. A spectrum was measured after reaching 95°C and then another time after allowing the sample to cool to 20°C again. For chemical denaturation, a cuvette with a pathlength of 1 cm was used. A constant volume of purified protein in PBS was mixed with varying amounts of PBS and denaturing buffer (7.73 M GdmCl solubilized in PBS; GdmCl from EMD Millipore) to give a range of denaturant concentrations and held at room temperature. GdmCl concentration was checked by measuring refractive index with a refractometer. After two days, we observed no change in CD signal compared to two hours after adding denaturant, and the signal at 222 nm after two days was used to plot the unfolding transition curve.

### 2.4. Protein Structure Determination with X-Ray Crystallography

Purified OT3 protein was concentrated to 7 mg/ml in 20 mM Tris, 150 mM NaCl, pH 8.0, and 0.15 *μ*L was mixed with an equal volume of crystallization buffer using the Morpheus screen. Crystals were grown using the sitting drop vapor in an incubator at 12°C. Crystallization experiments were set up using a Douglas Oryx8 Nanodrop dispensing robot (Douglas Instruments Ltd, Berkshire, United Kingdom). Within one week, many crystals appeared from several different crystallization conditions. We set up a focused screen centered on Morpheus well A10 where the best crystals seemed to appear (divalent metal ions, buffer Tris-Bicine, 10% PEG-8000, 20% ethylene glycol, pH 8.5). Crystals were harvested and cryocooled under liquid N_2_. In general, crystals harvested even from a single crystallization condition showed a huge variation in X-ray diffracting power, and therefore, a large number were screened for initial data quality assessment. Then, a full dataset collection was performed. Data collection to a minimum Bragg spacing of 1.92 Å was performed at 100 K using Stanford Synchrotron Radiation Lightsource (SSRL) beamline 9-2 (SLAC National Accelerator Laboratory, Menlo Park, CA, USA) [28]. Diffraction-quality crystals were obtained from a crystallization screening condition composed of 0.03 M MgCl_2_(H_2_O)_6_, 0.03 M CaCl_2_(H_2_O)_2_, 0.1 M Tris/Bicine, 10% PEG-8000, and 20% ethylene glycol at pH 8.5. The structure was solved by the molecular replacement method with Phaser (McCoy et al., 2007) using the OT3 design model as the search model, and the search was carried over the orthorhombic P222 crystal class to fix screw translations. The crystal belonged to the space group P 2_1_ 2_1_ 2_1_ and contained one polypeptide chain per asymmetric unit. Residues 1-228 were unambiguously traced in the electron density maps. Throughout refinement with PHENIX [29] manual adjustments on the polypeptide chain was made in Coot [30], trimming resolution from 1.92 Å to 2.1 Å. Solvent water molecules were first assigned based on their hydrogen bonding properties; in later stages of refinement, further water molecules were automatically added. Refinement progressed to convergence and reached strong agreement between the model and the experimental data. Data was reduced with XDS and scaled with SCALA [31, 32]. Hydrophobic cluster analysis was performed with ProteinTools [33], and packstat was computed with RosettaHoles [34, 35].

## 3. Results

### 3.1. De Novo Design Strategy

In an extensive survey of TIMB structures, Nagano et al. attributed the observed distortion in the shape of the barrel to residue packing patterns [36]. However, TIMB proteins in general and in particular the de novo TIMB are highly sensitive to sequence perturbations in the central barrel. Previous efforts to redesign or make mutations to the sequence to alter barrel eccentricity largely resulted in insoluble protein: these included introducing various mutations into the central barrel to force a change in curvature, rebuilding the structure with twofold symmetrical sequences, and changing the packing between the helices to induce flattened helical arrangements. Thus, a fully de novo approach to redesigning the shape of the barrel seemed necessary. We sought new design principles to achieve the design of a TIM barrel (TIMB) with elongated or “ovoid” *β* barrel curvature. Such a TIMB would ideally exhibit the maximum twofold symmetry while adhering to universal TIMB structural parameters, for example, the number of strands (n=8) and the shear number (s=8). Additionally, a de novo approach provides opportunities to optimize structural elements and facilitate downstream redesignability. For example, we wanted to replace the structural loops found in the de novo circular TIMB with loops that are more amenable to diverse conformations. We also wanted to increase the helix lengths and helix-sheet spacing to allow for increasing the buried hydrophobic surface area, which is known to contribute to protein stability [3, 37–40].

**Figure 1:**
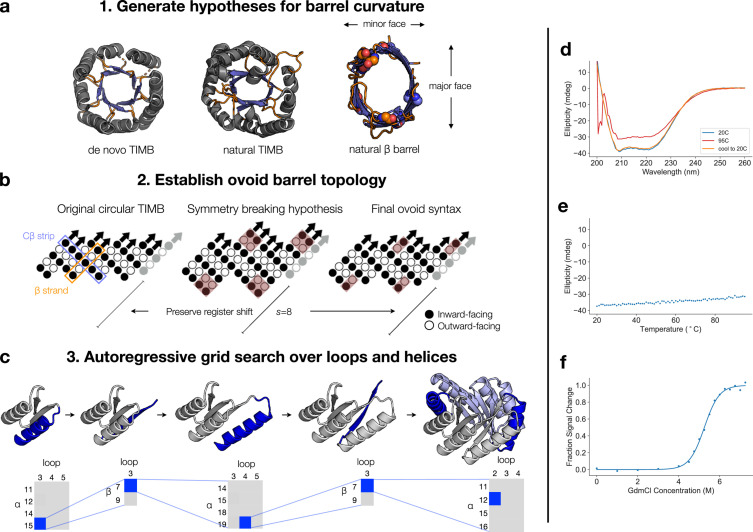
Design and characterization of ovoid TIM barrel. (a) Structural comparison of ovoid TIMBs and *β* barrels. Left: de novo TIMB (PDB 5BVL); middle: triose phosphate isomerase (PDB 1TIM); right: natural ovoid porin beta barrel (PDB 2WJQ). Loops are colored in orange, and beta strands are colored in blue. For the porin structure, glycine residues are indicated with spheres. (b) The *β* barrel strategy used to design the ovoid TIMB. The symmetry of the circular TIMB was broken by extending two pairs of strands to length nine; final solution after grid search yielded two pairs of extended strands of length seven. (c) The autoregressive sampling procedure used to generate the ovoid TIMB backbone. Top: grey indicates the constant portion of structure that is conditioned upon; blue indicates the variable portion of structure that is sampled. In the final frame, light blue is used to indicate the repeat structure which is constant during sampling. Bottom: in each phase, different combinations of loop and *α*/*β* lengths are sampled thoroughly and examined; the best solution is then fixed for the next phase. (d) CD spectra of OT1. A spectrum was taken at room temperature, then again after heating, then again after cooling back to room temperature. (e) Thermal heating of OT1 monitored by CD at 215 nm. (f) Chemical denaturation of OT1 measured by CD at 222 nm. Data was fit with a two-state transition model allowing for pre- and posttransition slopes.

Structural inspection of natural ovoid TIMBs and *β* barrels revealed two key observations that guided our design approach (Figure 1(a)). We refer to segments of barrels where the curvature is tighter (smaller radius of curvature) as the “minor face” and segments where the curvature is wider (greater radius of curvature) as the “major face.” The first was an enrichment of sterically smaller residues at the minor face along the “C*β* strips,” which line the interior of the barrel perpendicular to the strand direction (Figure 1(b); see reference [4] for the origin of this nomenclature). From this, we hypothesized that the smaller radius of curvature at the minor face is favored if the steric strain from the C*β* strips is minimized with smaller residues. A second inspiration came from observing the structure of NanC porin (PDB ID 2WJQ, Figure 1(a)). As a transmembrane pore, the porin *β* barrel naturally exhibits ovoid barrel geometry without needing a fully packed interior to sustain the eccentricity. We observed an enrichment of glycine (small) residues at the minor faces of NanC. Additionally, the flattened major face appears to correlate with the presence of the longest *β* strands in the structure. We recognize that this unique feature has not been described previously in the literature as a topologically derived mechanism to control *β* barrel geometry [23, 36], and this feature is even less accentuated in TIMBs because of their vastly diverse barrel geometry and relatively short *β* strands. A post facto inspection of native TIMB structures revealed a potential agreement with the long-strand scheme. This second design hypothesis, in which we enrich for longer *β* strands along the major or elongated axis of the barrel, guided our modification of the syntax from that of the earlier circular de novo TIMB (Figure 1(b)). Since the horizontal translation of the barrel is in part determined by the length of the strands in that region of the barrel, we hypothesized that longer strands enable more elongated barrel dimensions along the corresponding face/axis.

From these two hypotheses, we constructed a new blueprint for ovoid TIMB design (Figure 1(b)). Working from the original sTIM11 syntax with four pairs of parallel strands [15], we broke symmetry by extending the length of two alternating pairs of strands from five to nine residues. The register shift was adjusted so that the barrel shifted four residues between each repeat, preserving the overall shear number, s=8. With the interior topology established under this hypothesis for the *β* barrel, we searched for the ideal helix and loop lengths to complement this new *β* barrel syntax. Since the full combinatorial space over potential helix and loop lengths was too complicated to search via Rosetta fragment sampling, we resorted to an autoregressive approach (Figure 1(c)). Beginning at the N terminus, each successive secondary structure element was sampled conditioned on the preceding elements and selected by Rosetta energy scoring and manual curation. We initiated the process with the repeat subunit from sTIM11, with two *β* strands of length five together with the linking helix and loops. Loop and helix lengths which enriched for ideal arrangement of the *α* helices relative to the sampled *β* strands were fixed. *αβ* turn lengths were fixed at three to favor selection of a common and stable loop motif in natural TIMBs. The final helix of the repeating subunit was sampled jointly in repeat mode to produce full twofold repeat TIMB models. In this final step, we found that assessing the *α*/*β* barrel likelihood under a protein domain classifier was an effective method for ranking plausible design models [25]. This classifier is a deep learning model that parameterizes a residue-wise distribution over all protein folds and enabled us to quickly enrich for TIMB-like designs. This autoregressive design process led us to alter the lengths of some strands and settle on a final blueprint with strands of length five, five, seven, and seven, with register shifts of two after the third and fourth strand (Figure 1(b)).

Sequences were designed in Rosetta using an iterative enrichment procedure in which the Monte Carlo search space was narrowed down in successive rounds of design, based on amino acids enriched, or more frequently selected by the design algorithm, at each position [15, 26]. During the sequence design process, we observed many different barrel eccentricities, suggesting that our syntax is amenable to more curvatures than the ones designed here (see Materials and Methods). We selected an initial optimized sequence and observed that alanine residues were automatically favored at the narrower segments of the *β* barrel, aligning with our earlier hypothesis for sterically smaller residues at these positions. To evaluate the designed sequence, ab initio structure prediction was performed to evaluate the folding landscape [27, 41]. While we did not observe idealized energy funnels, we did find that low energy models were able to obtain the correct fold and overall structure (Fig. S2D–F). For low energy models which did not obtain the correct structural organization or topology, we looked for hypotheses to improve the structural signal contained in the sequence. In some models, misplaced helices alerted us to the presence of several unsatisfied hydrogen bonding atoms in the first *β*-*α* loop (Fig. S2A). In other models, the third *β* strand was “flipped” such that residues designed to be center-facing were instead facing towards the helices, disrupting the orientation of the surrounding helices and the overall structure (Fig. S2B, C). To address the loop hydrogen bonding issue, we resampled the loop length and conformation and obtained a more idealized loop. To address the strand flip, we sought to stabilize the design conformation with H36K and K50H mutations (and their corresponding repeat mutations), intended to encourage a solvent-facing orientation at position 36 and form a specific hydrogen bond between position 50 and the *αβ*3 loop backbone. These changes resulted in design OT1 (Ovoid TIMB 1); we retained the original loop in design OT6 and the original “flipped strand” sequence (but included the new loop) in design OT5. We made other mutations to fine-tune solvent-exposed positions and hydrophobic packing, yielding designs OT2-4 (Table S1). The resulting designs exhibited slightly improved folding plots, suggesting that our modifications improved the folding landscape of the optimized designs (Fig. S2D–F).

### 3.2. Biophysical Validation of Computational Designs

We next sought to experimentally validate the designed ovoid TIMBs. Genes encoding the sequences were expressed in E. coli, and the resulting protein was purified by nickel affinity and size exclusion chromatography. All six designs expressed well, and a soluble monomeric form was obtained by size exclusion chromatography (Fig. S3A, B). The designs showed similar CD spectra characteristic of the secondary structures present in the ovoid TIMB design (Figure 1(d) and Fig. S4A). Unfolding experiments revealed the designs to be highly thermostable, remaining folded even at 95°C (Figure 1(e) and Fig. S4B). To measure the thermodynamic stability of the protein, we performed chemical denaturation of OT1 in guanidine hydrochloride (GdmCl). This confirmed the high stability of the protein, with a computed ΔG of folding of 8.4 kcal/mol (m=1.6, $C_{m}=5.25$) (Figure 1(f)). Together, these results suggested that we had identified an adequate syntax for the ovoid TIMB architecture and that the sequences designed on the designed backbone obtain the desired increased stability.

**Figure 2:**
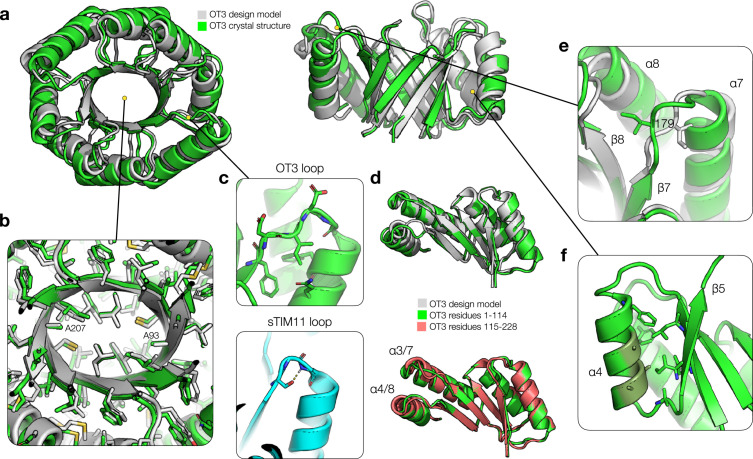
Crystal structure of OT3. (a) Comparison of OT3 design model (grey) and X-ray crystal structure (green). The top view is shown on the left, and the side view with helices 1-3 cut away is shown on the right. (b) Cutaway view of side chain packing in the hydrophobic core, with side chain heavy atoms shown as sticks. (c) Top: *βα* loop 3 is shown with side chains and backbone atoms and some surrounding side chains. Bottom: structural loop from sTIM11 is shown with core-facing Ser hydrogen bonded to a loop backbone atom. (d) Top: alignment of first half of OT3 structure to first half of design model. Bottom: alignment of first half of OT3 structure to second half of OT3 structure. (e) *βα* loop 7 shown with Ile179 packing in a different conformation compared to the design model. (f) An example of helical Ala residues forming less ideal hydrophobic cluster interactions and a lack of strong structural “knobs-into-holes” anchoring. Ala residues on *α*4 (dark green) and nearby side chains are shown.

In order to assess whether the proteins fold as designed, and especially whether the ovoid *β* barrel curvature is maintained, we determined the 3D structure of the OT3 design by X-ray crystallography using molecular replacement with the design model (Table S2). The experimental structure aligns with high accuracy to the design model, with an overall C*α* RMSD of 1.59 Å. In particular, the *β* barrel showed excellent fidelity, exhibiting just 0.74 Å RMSD over *α* and *β* carbon atoms and mirroring closely the ovoid curvature specified in the computational design (Figure 2(a)). Side chain packing in the hydrophobic regions also closely followed the designed rotamers. Especially, the close packing around A93 and A207 suggests that the placement of sterically small residues at the ovoid barrel minor face was a successful strategy (Figure 2(b)). The redesigned *β*-*α* loops are free of any critical hydrogen bonding or structural interactions as in the original sTIM11, enabling their redesign and diversification (Figure 2(c)) [17]. To assess the quality of the twofold symmetry design, we aligned the first repeat subunit to the second and found close agreement (0.74 Å C*α* RMSD), indicating that the designed twofold symmetry is self-consistent and viable (Figure 2(d)).

**Figure 3:**
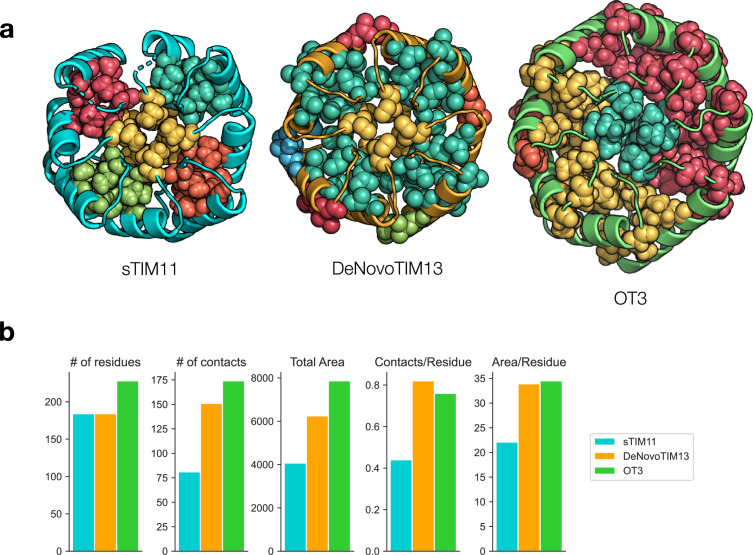
Analysis of hydrophobic clustering. (a) ILV hydrophobic clusters for sTIM11 (PDB ID 5BVL), DeNovoTIM13 (PDB ID 6YQX), and OT3 (PDB ID 7UEK). Each unique cluster is colored separately. Atoms (including hydrogens) belonging to a cluster are visualized as spheres. (b) Statistics of hydrophobic clusters as calculated with CSU algorithm in ProteinTools. Area is in units of Å^2^.

Deviations between the design model and crystal structure can be largely explained by shifting of the helices and loops and may point to potential targets for future redesign. We noticed two main points of deviation: disagreement between the fourth and eighth helices (*α*4 and *α*8, which are symmetry mates) and the design model and disagreement between the seventh helix (*α*7) and the design model (these can be observed in Figure 2(d)). In the region of *α*7, the overall rigid-body structures of the seventh *β* strand (*β*7) and *α*7 are in agreement with the design model. However, residues from D178 to E182 adopt a different conformation from the design, likely driven by the packing of I179 into a void space near the eighth *β*-*α* turn (Figure 2(e)). Another explanation for the lack of structural constrains could be the RosettaRemodel fragment assembly scheme, which typically favors standard *β*-*α* connections but occasionally borrows fragments that align poorly to context. The altered loop conformation, combined with weakened “knobs-into-holes” style packing due to the presence of smaller A192 and A223 residues, amplifies the small differences in the *β* strands and is a potential reason for the displacement of *α*7 relative to the design model. The issue with alanine residues in interfacial packing is illustrated as well by the placements of *α*4 and *α*8, where three alanine residues form the majority of the interface with the *β* barrel (Figure 2(f)). In this design scenario, this reduces the size and strength of hydrophobic clusters and provides weaker structural anchoring for the helix relative to a larger residue. In another example, the N-terminal regions of the third and seventh *β* strands do not seem to form idealized secondary structure. This could be due to the fact that one of the +2 strand register shifts occurs between these strands and the following strands, which halves the number of hydrogen bonding interactions involved in secondary structure formation. This could be compounded by the weaker hydrophobic packing on both the inward and outward faces of the *β* barrel, which also represents a potential avenue for improvement. However, this structural behavior is not uncommon in natural TIMBs and exists also in the circular de novo TIMBs (i.e., sTIM11 and the DeNovoTIMs, see also Figure 3(a)).

Further analysis of the crystal structure suggests the importance of design decisions in achieving improved stability. Critically, lengthening the helices and increasing their spacing from the *β* barrel allows for increased burial of hydrophobic residues. The burial of isoleucine, leucine, and valine residues in “ILV” hydrophobic clusters has been hypothesized to be important for protein and especially TIMB stability [42–45]. Analysis of ILV hydrophobic clusters (using the CSU algorithm implemented in ProteinTools [33, 46]) in OT3 as compared with the circular sTIM11 indicates the presence of more tightly connected clusters: one cluster in the core of the *β* barrel, one cluster covering half of the helix-barrel packing interactions, and two clusters in the other half (Figure 3(a)). The total hydrophobic contacts and total buried area across all clusters are increased significantly in OT3 (174 contacts; 7864.8 Å^2^) compared to sTIM11 (81 contacts; 4067.8 Å^2^). These trends mirror those found in the DeNovoTIMs, which also exhibit consolidated clusters, increased buried contacts and area, and increased stability relative to sTIM11 (Figure 3(b)) [16]. The consistency of normalized burial surface area per contact (45.2 Å^2^/contact for OT3 vs. 50.2 Å^2^/contact for sTIM11) as well as packing quality (packstat=0.60 for OT3 and packstat=0.67 for sTIM11) across both the circular and ovoid TIMBs also suggests that the quantity rather than the quality of hydrophobic interactions may be more important for the increased stability. However, visual inspection reveals that previous flaws with sTIM11 are eliminated in OT3, such as a cavity in the central *β* barrel and gaps between each of the hydrophobic clusters (Figure 3(a)). We note that the ovoid curvature is an important factor in resolving the core cavity—with the center of the barrel at a varying radius from the surrounding *β* barrel, a greater variety of amino acids can be sampled to reach the center, whereas with a circular *β* barrel, the distance is both constant and greater (from most barrel positions), limiting the space of possible packing solutions. This is further supported by the observation that these gaps are reduced but not eliminated in the DeNovoTIMs (Figure 3(a)).

### 3.3. Stability and Robustness to Scaffold Perturbation

Since many functional sites in proteins contain polar and charged residues, and often in solvent-occluded pockets, the ability to introduce similar residue identities and pockets into designed proteins without causing excessive destabilization or unfolding is important for designing functional proteins. The high stability of the OT proteins led us to investigate the robustness of these scaffolds to destabilizing mutations, which might provide information on their viability as scaffolds for functional design. We designed two sets of mutants: one with buried polar or charged residues in the core and one ablating different regions of hydrophobic packing by mutating them to alanine. Since functional mutations are often destabilizing [47, 48], our rationale was to select these mutations to be at least as disruptive as those which might be designed while installing a new function.

**Figure 4:**
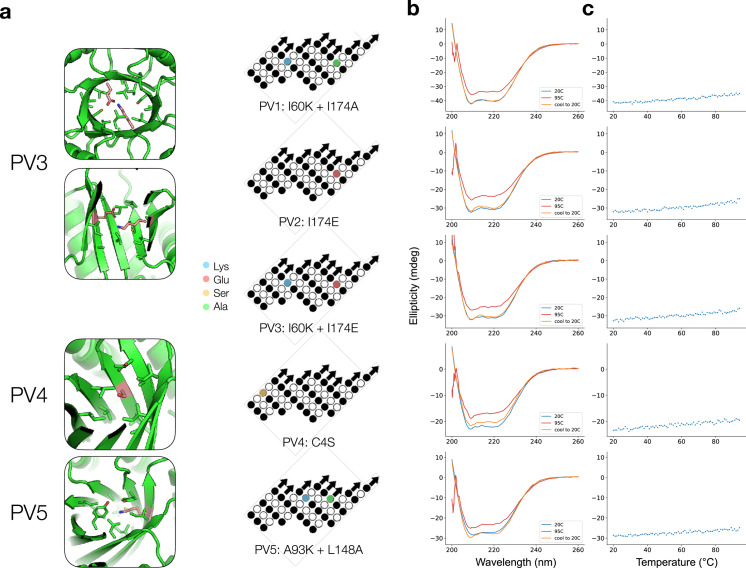
Characterization of polar variant designs. (a) Structural contexts of polar variant mutations. Left: mutations to ionizable residues are highlighted in pink in the structural diagrams; for PV3, each individual mutation is equivalent to either PV1 or PV2, so only PV3 is depicted. Right: mutations are indicated on the *β* barrel topology map from Figure 1(b) CD spectra of polar variants. Spectra were taken at room temperature, then again after heating, then again after cooling back to room temperature. Spectra correspond to the designs indicated by topology maps in (a). (c) Thermal heating of polar variants monitored at 209 nm for PV1-4 and at 210 nm for PV5.

For the polar/charged mutations, we mutated either one or two core residues to one of lysine, glutamate, or serine. PV1 (Polar Variant 1) has an I60K mutation with a sterically compensating I174A mutation in the same position on the opposite repeat subunit; PV2 has an I174E mutation; and PV3 represents the combination of these two (I60K and I174E) and is modeled as a buried salt bridge (Figure 4(a)). PV4 has a C4S mutation and PV5 pairs a A93K mutation with a sterically compensating L148A mutation (Figure 4(a)). PV1-2 and PV5 are intended to investigate the effect of burying a charged residue in different parts of the protein, while PV3 examines the effect of pairing a buried negative charge with a positive one. PV4 is intended as a more moderate mutation.

The polar variants were expressed and purified as before and found to exhibit highly similar CD profiles to the original OT proteins, indicating that the mutations likely do not cause major structural rearrangements, as sometimes occurs with burial of charged residues (Figure 4(b)) [49, 50]. Remarkably, despite the placement of charged residues in the hydrophobic core, the proteins all retained their high thermal stability, remaining folded even at 95°C, and any loss of secondary structure was recovered upon cooling (Figures 4(b) and 4(c)). However, we did note that the yield of expressed and purified protein was lower, suggesting that the mutations may have partially disrupted folding pathways and led to more misfolded protein.

**Figure 5:**
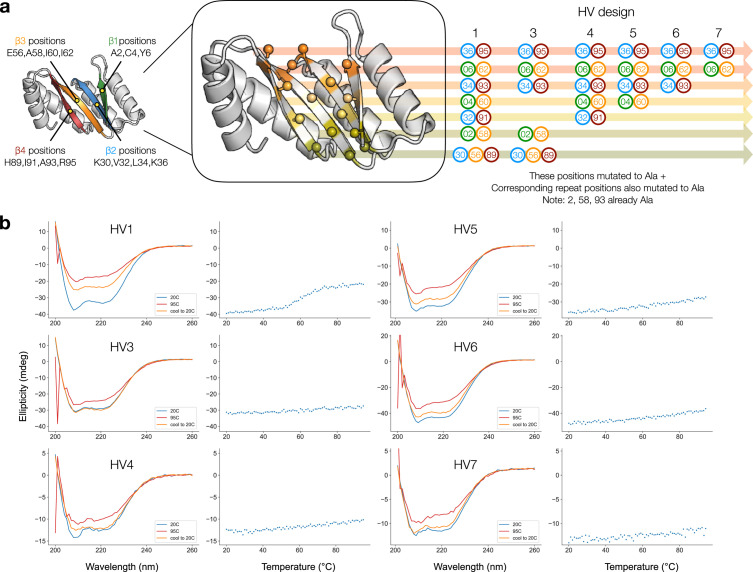
Characterization of hollow variant designs. (a) Illustration of TIMB “layers” and ablation mutations made to generate hollow variants. Left: a cutaway of the first repeat of the OT1 design model (residues 1-114) is shown with beta strands labeled and the relevant residue positions belonging to each strand. Center: the same cutaway is shown with Ca-Cb residue positions indicated as ball and stick; each layer is colored uniquely. Right: sets of layers are mutated to alanine to yield the HV designs, with specific positions listed. All residue numberings indicate only those in the first repeat, but for all HV designs, the corresponding residue in the second repeat was also mutated to alanine (e.g., if residue 2 was mutated, then residue 116 was also mutated; if residue 4 was mutated; then residue 118 was also mutated; and so on). (b) CD spectra and thermal heating of hollow variants monitored at 208 nm.

In a second set of variants, HV1 through HV7 (Hollow Variant), we mutated different “layers” of the hydrophobic core to alanine to simulate the effect of carving a binding pocket out of the core. Each layer is formed by alternating strands of the *β* barrel and describes a set of potentially interacting residues lying in a plane perpendicular to the axis of the *β* barrel [51]. Since the register shift is, on average, one residue per *β* strand, the core-facing positions of alternating *β* strands align to form horizontal layers through the core of the *β* barrel. For HV1 and HV4-7, the different designs represent crown pockets of decreasingly fewer mutations (Figure 5(a)). For HV3, we investigated whether mutations near the bottom of the barrel could also be made to install functional sites in that region (Figure 5(a)).

We expressed and purified these proteins and found that all but HV1 purified as soluble monomers. HV1 eluted at an earlier volume during size exclusion chromatography and appeared to purify in a dimeric form (Fig. S3B). We measured their CD spectra, and they exhibited the expected spectrum, similar to that of OT1 (Figure 5(b)). All variants except HV1 remained folded at 95°C, regardless of the number of alanine mutations made to the core (Figure 5(b)). HV1, which was the most severe hollow variant, with every single core residue mutated to alanine, still exhibited CD signal corresponding to residual secondary structure at high temperature. The partially irreversible reduction in helicity shown with a transition near 60°C may suggest that the unfolding kinetics for this variant is not a simple two-state. As with the polar variants, we found the hollow variants to yield less soluble protein (Fig. S3B). The unusual stability of these proteins, despite the ablation of core packing, suggests that hydrophobic packing in the region between the helices and *β* barrel can also be sufficient for high thermal stability. Furthermore, we expect these mutations to be far more drastic than typical mutations made to install functional motifs such as active sites, which may involve ablating only a few hydrophobic packing residues and installing only partially occluded ionizable residues rather than the mutations we have tested here.

## 4. Discussion

This work illustrates the potential of deriving simple structural hypotheses from studying natural proteins that can be implemented through de novo protein design. By uncovering design principles behind the curvature of the core *β* barrel in TIMBs, we have increased the accessible structural space of the original de novo TIMB and developed it into a powerful scaffold for various protein engineering and design efforts. The computational “flexibility” of the barrel curvature observed in design suggests that our new syntax could be used to explore differing degrees of eccentricity and enable fine control over TIMB curvature. In comparison to earlier de novo protein design studies, where large numbers of designs were tested with a success rate ranging from 25 to 40 percent, our high rate of soluble, stable expression (100 percent) among only a small set of experimentally tested designs suggests that our understanding of protein design has matured. However, as evidenced by the reduced yield of some designs, further work is needed to understand the dynamic process of protein folding and how sequence determines the traversal of the folding energy landscape.

Our work suggests ways in which de novo design can expand beyond the natural framework of evolution for generating new proteins. In natural TIMB proteins, stability appears to be driven by hydrophobic packing in the core region [21]. However, our de novo TIMB exhibits high stability even when these core residues are mutated away to alanine, indicating a unique mechanism for TIMB stability [52]. Furthermore, installing two interacting ionizable side chains simultaneously into the hydrophobic protein core is a simple and viable procedure in the de novo ovoid TIMB. In contrast, for a natural protein, this would involve destabilizing stepwise mutations and a highly unlikely evolutionary event [53, 54]. Indeed, when computational design is used to install sets of polar or charged residues into the interior of natural proteins, it is not uncommon for the resulting sequences to fail to express [55]. This demonstrates the potential of de novo design for achieving new functions and chemistries not explored in natural evolution.

The ability to support one or more polar amino acid at different positions in the core of the protein is important for many different functional applications. Hydrogen bond networks (buried and unburied) are important in molecular recognition and enzyme catalysis, conferring increased affinity, interaction specificity, charge stabilization, pK_a_ modulation, and other effects. The regularly placed and oriented *β* barrel positions in the de novo TIMB, which our results suggest may be able to assume any combination of amino acid identities, present a unique and powerful system in which to design these hydrogen bond networks–almost like a molecular “breadboard.” We anticipate that this system will be a useful scaffold in which many different functional proteins can be designed.

## Data Availability

Structure factors and coordinates have been deposited in the Protein Data Bank under accession code 7UEK. Design models from Rosetta are provided in Supplementary Materias. Further data are available from the corresponding author upon reasonable request.

## Supplementary Materials

Supplementary Materials The supplementary information contains further details about the design and characterization of the de novo proteins in this study, as well as Rosetta design models.
