# Supplementary material for: De Novo Design of a Highly Stable Ovoid TIM Barrel: Unlocking Pocket Shape towards Functional Design
Source: Biodes Res. 2022 Oct 10;2022:9842315. doi: 10.34133/2022/9842315 (PMC10521652; doi:10.34133/2022/9842315)
Supplement: Supplementary Materials — The supplementary information contains further details about the design and characterization of the de novo proteins in this study, as well as Rosetta design models. [file 9842315.f1.zip › Supplementary_Materials/supplementary_information.pdf]

SUPPLEMENTARY INFORMATION FOR

# De novo design of a highly stable ovoid TIM barrel: unlocking pocket shape towards functional design

Alexander E. Chu, Daniel Fernandez, Jingjia Liu, Raphael R. Eguchi, and Po-Ssu Huang

Example flags and blueprint (early round)

```
-s lb2_66_nochain.pdb
-remodel:blueprint design.bp
-jd2:no_output
-overwrite
-num_trajectory 1
-save_top 1
-repeat_structure 2
-chain _
-hb_lrbb 4.0
-constraints:cst_file lockup_cst.txt
-remodel:cstfilter 1000
-randomize_loops false
-bypass_fragments
-bypass_closure
-remodel:use_pose_relax
-remodel:dr_cycles 5
-soft_rep_design
-no_optH false
-ex1
-ex2
-relax:constrain_relax_to_start_coords
-score:set_weights coordinate_constraint 0.5
```

```
1 Q L PIKAA A
2 C L ALLAAxc
3 L L APOLAR
4 L L APOLAR
5 I L APOLAR
6 V L ALLAAxc
7 V L ALLAAxc
8 T L PIKAA A
9 G L ALLAAxc
10 D L PIKAA A
11 A L ALLAAxc
12 E L PIKAA A
13 D L ALLAAxc
14 L L APOLAR
15 K L PIKAA A
16 A L ALLAAxc
17 W L APOLAR
18 L L APOLAR
19 E L PIKAA A
20 I L ALLAAxc
.
.
.
95 E L PIKAA A
96 E L PIKAA A
97 E L PIKAA A
98 D L PIKAA A
99 K L PIKAA A
100 A L APOLAR
101 R L PIKAA A
102 K L PIKAA A
103 N L ALLAAxc
104 L L APOLAR
105 K L PIKAA A
106 I L ALLAAxc
107 A L APOLAR
108 L L ALLAAxc
109 D L PIKAA A
110 L L ALLAAxc
111 G L PIKAA A
112 V L APOLAR
```

Example flags and blueprint (late round)

```
-s r2d5.pdb
-remodel:blueprint restrict_polar_repack.bp
-jd2:no_output
-overwrite
-num_trajectory 1
-save_top 1
-repeat_structure 2
-chain _
-hb_lrbb 4.0
-constraints:cst_file lockup_cst.txt
-remodel:cstfilter 1000
-randomize_loops false
-bypass_fragments
-bypass_closure
-remodel:use_pose_relax
-remodel:dr_cycles 3
-no_optH false
-ex1
-ex2
```

```
1 K L PIKAA D
2 A L PIKAA A
3 I L PIKAA I
4 C L PIKAA C
5 I L PIKAA I
6 I L PIKAA I
7 L L PIKAA LV
8 D L PIKAA DKETNR
9 G L PIKAA GS
10 D L PIKAA D
11 W L PIKAA W
12 K L PIKAA KER
13 D L PIKAA DEQVM
14 M L PIKAA M
15 E L PIKAA KER
16 K L PIKAA KRND
17 A L PIKAA A
18 M L PIKAA M
19 K L PIKAA EKRH
20 I L PIKAA I
.
.
.
95 R L PIKAA KREDQTN
96 S L PIKAA S
97 D L PIKAA DEKQS
98 E L PIKAA ED
99 Q L PIKAA KEDTQ
100 F L PIKAA F
101 K L PIKAA KR
102 R L PIKAA KR
103 L L PIKAA LM
104 A L PIKAA A
105 K L PIKAA EK
106 I L PIKAA I
107 A L PIKAA A
108 A L PIKAA ARLY
109 E L PIKAA ERK
110 L L PIKAA L
111 G L PIKAA G
112 A L PIKAA A
```

Beta barrel constraints

```
AtomPair N 28 O 1 HARMONIC 2.8 0.4
AtomPair N 3 O 28 HARMONIC 2.8 0.4
AtomPair N 30 O 3 HARMONIC 2.8 0.4
AtomPair N 5 O 30 HARMONIC 2.8 0.4
AtomPair N 32 O 5 HARMONIC 2.8 0.4
AtomPair N 54 O 27 HARMONIC 2.8 0.4
AtomPair N 29 O 54 HARMONIC 2.8 0.4
AtomPair N 56 O 29 HARMONIC 2.8 0.4
AtomPair N 31 O 56 HARMONIC 2.8 0.4
AtomPair N 58 O 31 HARMONIC 2.8 0.4
AtomPair N 33 O 58 HARMONIC 2.8 0.4
AtomPair N 87 O 55 HARMONIC 2.8 0.4
AtomPair N 57 O 87 HARMONIC 2.8 0.4
AtomPair N 89 O 57 HARMONIC 2.8 0.4
AtomPair N 59 O 89 HARMONIC 2.8 0.4
AtomPair N 91 O 59 HARMONIC 2.8 0.4
AtomPair N 61 O 91 HARMONIC 2.8 0.4
AtomPair N 114 O 88 HARMONIC 2.8 0.4
AtomPair N 90 O 114 HARMONIC 2.8 0.4
AtomPair N 116 O 90 HARMONIC 2.8 0.4
AtomPair N 92 O 116 HARMONIC 2.8 0.4
AtomPair N 118 O 92 HARMONIC 2.8 0.4
AtomPair N 140 O 113 HARMONIC 2.8 0.4
AtomPair N 115 O 140 HARMONIC 2.8 0.4
AtomPair N 142 O 115 HARMONIC 2.8 0.4
AtomPair N 117 O 142 HARMONIC 2.8 0.4
AtomPair N 144 O 117 HARMONIC 2.8 0.4
AtomPair N 166 O 139 HARMONIC 2.8 0.4
AtomPair N 141 O 166 HARMONIC 2.8 0.4
AtomPair N 168 O 141 HARMONIC 2.8 0.4
AtomPair N 143 O 168 HARMONIC 2.8 0.4
AtomPair N 170 O 143 HARMONIC 2.8 0.4
AtomPair N 199 O 167 HARMONIC 2.8 0.4
AtomPair N 169 O 199 HARMONIC 2.8 0.4
AtomPair N 201 O 169 HARMONIC 2.8 0.4
AtomPair N 171 O 201 HARMONIC 2.8 0.4
AtomPair N 203 O 171 HARMONIC 2.8 0.4
AtomPair N 173 O 203 HARMONIC 2.8 0.4
AtomPair N 205 O 173 HARMONIC 2.8 0.4
AtomPair N 2 O 200 HARMONIC 2.8 0.4
AtomPair N 202 O 2 HARMONIC 2.8 0.4
AtomPair N 4 O 202 HARMONIC 2.8 0.4
AtomPair N 204 O 4 HARMONIC 2.8 0.4
AtomPair N 6 O 204 HARMONIC 2.8 0.4
```

**Figure S1. Arguments and blueprints for Rosetta design**

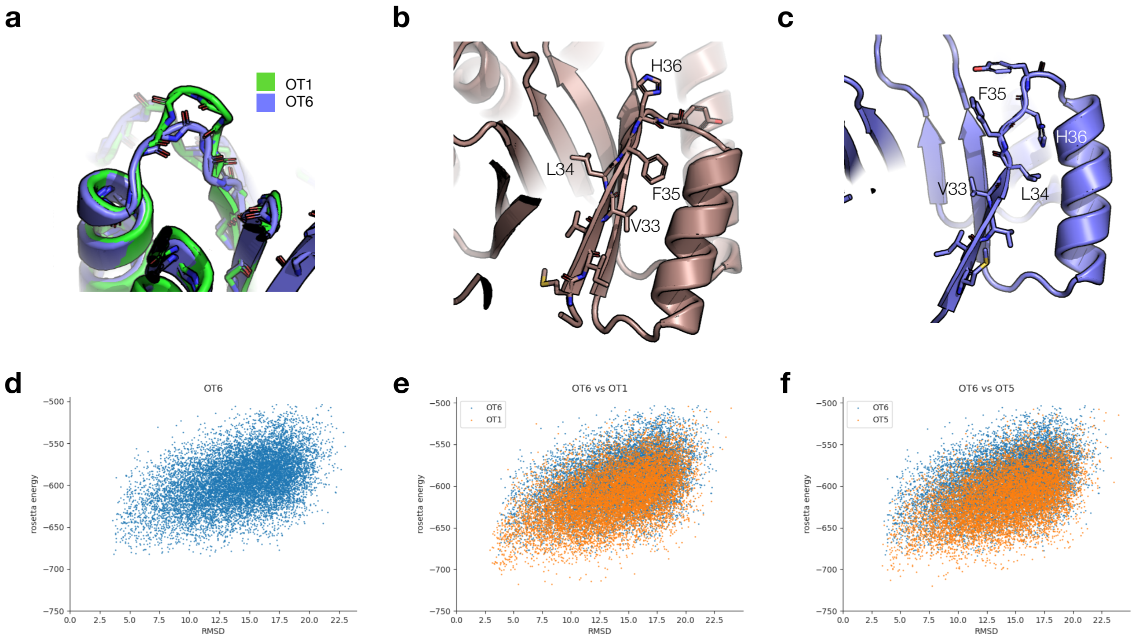


**Figure S2. Ab initio structure prediction and analysis of OT6, OT1 and OT5**
a) Comparison of loop 1 backbone conformations for designs OT1 and OT6.
b) The OT5 design model showing the intended helix-facing residues (V33, F35) and the center-facing residues (L34, H36). Note that of these positions only H36 is fully exposed to solvent.
c) An ab initio predicted structure showing the aberrant "flipped" strand prediction with L34, H36 helix-facing and V33, F35 center-facing.
d) Ab initio structure prediction folding trajectories for OT6.
e) Ab initio structure prediction folding trajectories; comparison between OT6 (blue) and OT1 (orange).
f) Ab initio structure prediction folding trajectories; comparison between OT6 (blue) and OT5 (orange).

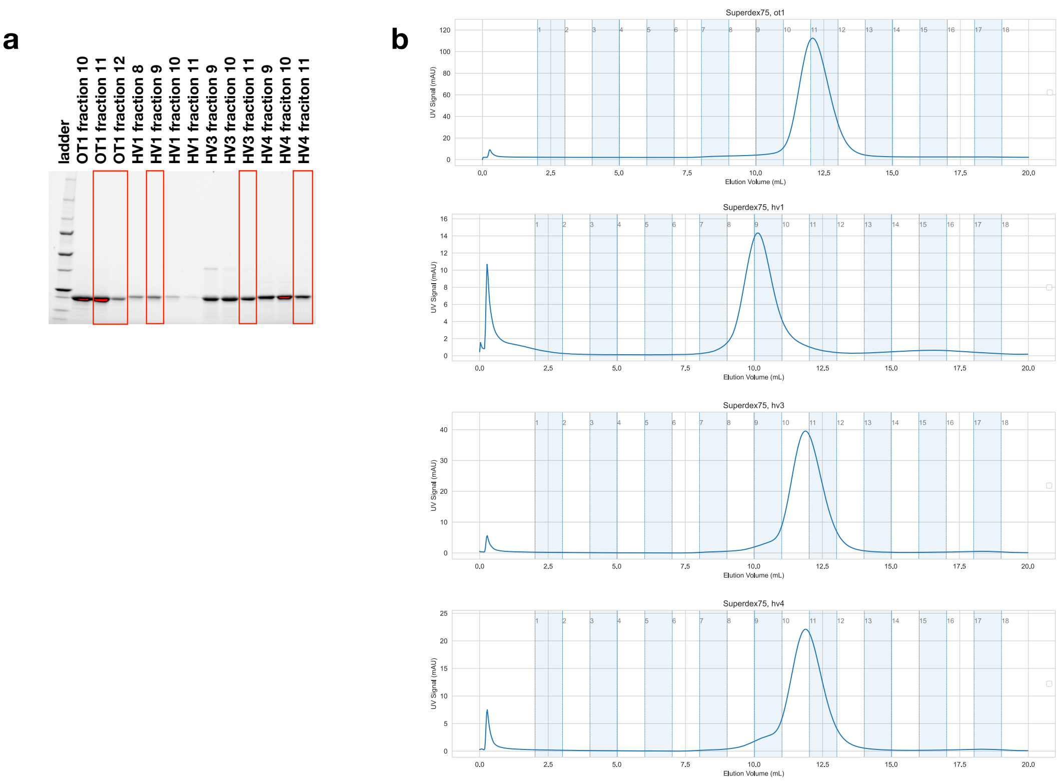


**Figure S3. Purification of ovoid TIMB designs and mutants**

a) After size exclusion chromatography, selected fractions were chosen for analysis by SDS-PAGE. All designs except HV1, HV3, and HV4 purified as and behaved similarly to OT1 and data for OT1 is representative of these designs. We further analyzed HV1, HV3, and HV4 since these showed a greater propensity to aggregate prior to purification and exhibited more diverse SEC chromatograms. The fractions highlighted in red were used for downstream biophysical experiments and (except for OT1 fraction 12) for the analytical SEC traces in (b).

b) Analytical size exclusion chromatography traces for OT1 fraction 11, HV1 fraction 9, HV3 fraction 11, and HV4 fraction 11 from (a).

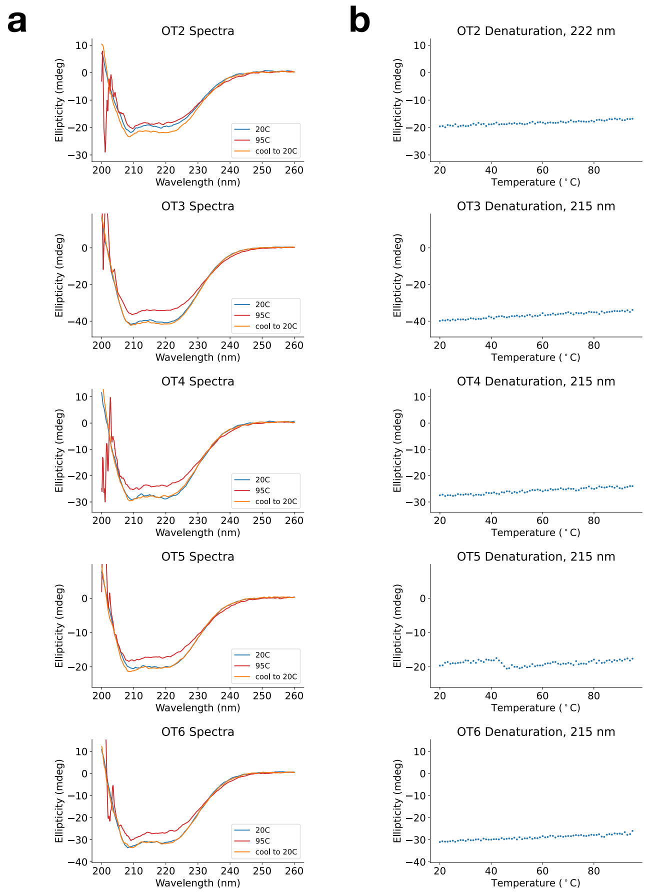


**Figure S4. Folding and thermal stability of OT2-6 designs**

a) CD spectra for OT2-6 at 20 °C, 95 °C, and at 20 °C again after cooling.

b) CD signal at indicated wavelength during thermal heating to 95 °C for OT2-6.

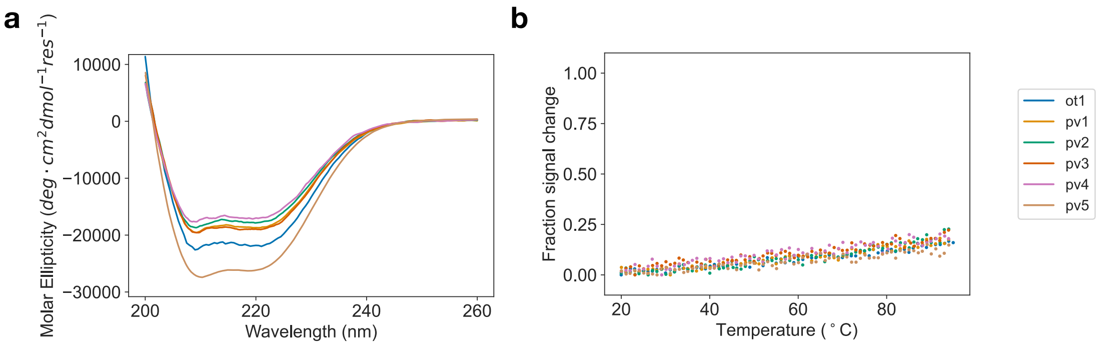


**Figure S5. Comparison of PV folding and stability with OT1**

a) Comparative plot of CD spectra for OT1 and the polar variant designs. (Data are the same as those shown in Fig. 4).

b) Comparative plot of thermal denaturation for OT1 and the polar variant designs. (Data are the same as those shown in Fig. 4).

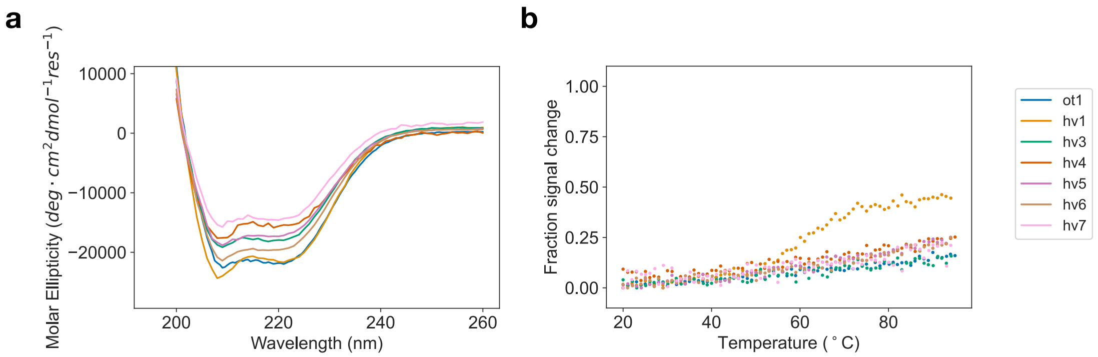


**Figure S6. Comparison of HV folding and stability with OT1**

a) Comparative plot of CD spectra for OT1 and the hollow variant designs. (Data are the same as those shown in Fig. 5).

b) Comparative plot of thermal denaturation for OT1 and the hollow variant designs. (Data are the same as those shown in Fig. 5).

## Table S1. Sequences of ovoid TIMB designs and mutants

| Design | Description | Sequence |
|---|---|---|
| OT1 | consensus | DAICIYLDESATWKDMKKAMEILYKLGVKKIVVLFKYDEKLIKVAAKVLHDLGAEEAAIILIFDIDDEDEFKKQVKKAL<br>ELMKKLGVDHRIIALRMTDEEKFKKLAKIAAELGADAICIYLDESATWKDMKKAMEILYKLGVKKIVVLFKYDEKLIKV<br>AAKVLHDLGAEEAAIILIFDIDDEDEFKKQVKKALELMKKLGVDHRIIALRMTDEEKFKKLAKIAAELGA |
| OT2 | Y6A (and repeat mutation) | DAICIALDESATWKDMKKAMEILYKLGVKKIVVLFKYDEKLIKVAAKVLHDLGAEEAAIILIFDIDDEDEFKKQVKKAL<br>ELMKKLGVDHRIIALRMTDEEKFKKLAKIAAELGADAICIALDESATWKDMKKAMEILYKLGVKKIVVLFKYDEKLIKV<br>AAKVLHDLGAEEAAIILIFDIDDEDEFKKQVKKALELMKKLGVDHRIIALRMTDEEKFKKLAKIAAELGA |
| OT3 | A58I (and repeat mutation) | DAICIYLDESATWKDMKKAMEILYKLGVKKIVVLFKYDEKLIKVAAKVLHDLGAEEAIIILIFDIDDEDEFKKQVKKAL<br>ELMKKLGVDHRIIALRMTDEEKFKKLAKIAAELGADAICIYLDESATWKDMKKAMEILYKLGVKKIVVLFKYDEKLIKV<br>AAKVLHDLGAEEAIIILIFDIDDEDEFKKQVKKALELMKKLGVDHRIIALRMTDEEKFKKLAKIAAELGA |
| OT4 | K30M, E56Y, H89I (and repeat mutations) | DAICIYLDESATWKDMKKAMEILYKLGVKMIVVLFKYDEKLIKVAAKVLHDLGAEYAAIILIFDIDDEDEFKKQVKKAL<br>ELMKKLGVDIRIIALRMTDEEKFKKLAKIAAELGADAICIYLDESATWKDMKKAMEILYKLGVKMIVVLFKYDEKLIKV<br>AAKVLHDLGAEYAAIILIFDIDDEDEFKKQVKKALELMKKLGVDIRIIALRMTDEEKFKKLAKIAAELGA |
| OT5 | OT4 + K36H, H50K (and repeat mutations) | DAICIYLDESATWKDMKKAMEILYKLGVKMIVVLFHYDEKLIKVAAKVLKDLGAEYAAIILIFDIDDEDEFKKQVKKAL<br>ELMKKLGVDIRIIALRMTDEEKFKKLAKIAAELGADAICIYLDESATWKDMKKAMEILYKLGVKMIVVLFHYDEKLIKV<br>AAKVLKDLGAEYAAIILIFDIDDEDEFKKQVKKALELMKKLGVDIRIIALRMTDEEKFKKLAKIAAELGA |
| OT6 | remodel ba1 and ba5 loops | DAICIILDGDWKDMKKAMEILYKLGVKKIVVLFKYDEKLIKVAAKVLHDLGAEEAAIILIFDIDDEDEFKKQVKKALEL<br>MKKLGVDHRIIALRLKSDEKFKKLAKIAAELGADAICIILDGDWKDMKKAMEILYKLGVKKIVVLFKYDEKLIKVAAKV<br>LHDLGAEEAAIILIFDIDDEDEFKKQVKKALELMKKLGVDHRIIALRLKSDEKFKKLAKIAAELGA |
| PV1 | I60K, I174A | DAICIYLDESATWKDMKKAMEILYKLGVKKIVVLFKYDEKLIKVAAKVLHDLGAEEAAIKLIFDIDDEDEFKKQVKKAL<br>ELMKKLGVDHRIIALRMTDEEKFKKLAKIAAELGADAICIYLDESATWKDMKKAMEILYKLGVKKIVVLFKYDEKLIKV<br>AAKVLHDLGAEEAAIALIFDIDDEDEFKKQVKKALELMKKLGVDHRIIALRMTDEEKFKKLAKIAAELGA |
| PV2 | I174E | DAICIYLDESATWKDMKKAMEILYKLGVKKIVVLFKYDEKLIKVAAKVLHDLGAEEAAIILIFDIDDEDEFKKQVKKAL<br>ELMKKLGVDHRIIALRMTDEEKFKKLAKIAAELGADAICIYLDESATWKDMKKAMEILYKLGVKKIVVLFKYDEKLIKV<br>AAKVLHDLGAEEAAIELIFDIDDEDEFKKQVKKALELMKKLGVDHRIIALRMTDEEKFKKLAKIAAELGA |
| PV3 | I60K, I174E | DAICIYLDESATWKDMKKAMEILYKLGVKKIVVLFKYDEKLIKVAAKVLHDLGAEEAAIKLIFDIDDEDEFKKQVKKAL<br>ELMKKLGVDHRIIALRMTDEEKFKKLAKIAAELGADAICIYLDESATWKDMKKAMEILYKLGVKKIVVLFKYDEKLIKV<br>AAKVLHDLGAEEAAIELIFDIDDEDEFKKQVKKALELMKKLGVDHRIIALRMTDEEKFKKLAKIAAELGA |
| PV4 | C4S | DAISIYLDESATWKDMKKAMEILYKLGVKKIVVLFKYDEKLIKVAAKVLHDLGAEEAAIILIFDIDDEDEFKKQVKKAL<br>ELMKKLGVDHRIIALRMTDEEKFKKLAKIAAELGADAICIYLDESATWKDMKKAMEILYKLGVKKIVVLFKYDEKLIKV<br>AAKVLHDLGAEEAAIILIFDIDDEDEFKKQVKKALELMKKLGVDHRIIALRMTDEEKFKKLAKIAAELGA |
| PV5 | A93K, L148A | DAICIYLDESATWKDMKKAMEILYKLGVKKIVVLFKYDEKLIKVAAKVLHDLGAEEAAIILIFDIDDEDEFKKQVKKAL<br>ELMKKLGVDHRIIKLRMTDEEKFKKLAKIAAELGADAICIYLDESATWKDMKKAMEILYKLGVKKIVVAFKYDEKLIKV<br>AAKVLHDLGAEEAAIILIFDIDDEDEFKKQVKKALELMKKLGVDHRIIALRMTDEEKFKKLAKIAAELGA |
| HV1 | see Fig. 4A | DAIAIALDESATWKDMKKAMEILYKLGVKAIAVAFAYDEKLIKVAAKVLHDLGAEAAAIALAFDIDDEDEFKKQVKKAL<br>ELMKKLGVDARAIALAMTDEEKFKKLAKIAAELGADAIAIALDESATWKDMKKAMEILYKLGVKAIAVAFAYDEKLIKV<br>AAKVLHDLGAEAAAIALAFDIDDEDEFKKQVKKALELMKKLGVDARAIALAMTDEEKFKKLAKIAAELGA |
| HV3 | see Fig. 4A | DAICIALDESATWKDMKKAMEILYKLGVKAIVVAFAYDEKLIKVAAKVLHDLGAEAAAIILAFDIDDEDEFKKQVKKAL<br>ELMKKLGVDARIIALAMTDEEKFKKLAKIAAELGADAICIALDESATWKDMKKAMEILYKLGVKAIVVAFAYDEKLIKV<br>AAKVLHDLGAEAAAIILAFDIDDEDEFKKQVKKALELMKKLGVDARIIALAMTDEEKFKKLAKIAAELGA |
| HV4 | see Fig. 4A | DAIAIALDESATWKDMKKAMEILYKLGVKKIAVAFAYDEKLIKVAAKVLHDLGAEEAAIALAFDIDDEDEFKKQVKKAL<br>ELMKKLGVDHRAIALAMTDEEKFKKLAKIAAELGADAIAIALDESATWKDMKKAMEILYKLGVKKIAVAFAYDEKLIKV<br>AAKVLHDLGAEEAAIALAFDIDDEDEFKKQVKKALELMKKLGVDHRAIALAMTDEEKFKKLAKIAAELGA |
| HV5 | see Fig. 4A | DAIAIALDESATWKDMKKAMEILYKLGVKKIVVAFAYDEKLIKVAAKVLHDLGAEEAAIALAFDIDDEDEFKKQVKKAL<br>ELMKKLGVDHRIIALAMTDEEKFKKLAKIAAELGADAIAIALDESATWKDMKKAMEILYKLGVKKIVVAFAYDEKLIKV<br>AAKVLHDLGAEEAAIALAFDIDDEDEFKKQVKKALELMKKLGVDHRIIALAMTDEEKFKKLAKIAAELGA |
| HV6 | see Fig. 4A | DAICIALDESATWKDMKKAMEILYKLGVKKIVVAFAYDEKLIKVAAKVLHDLGAEEAAIILAFDIDDEDEFKKQVKKAL<br>ELMKKLGVDHRIIALAMTDEEKFKKLAKIAAELGADAICIALDESATWKDMKKAMEILYKLGVKKIVVAFAYDEKLIKV<br>AAKVLHDLGAEEAAIILAFDIDDEDEFKKQVKKALELMKKLGVDHRIIALAMTDEEKFKKLAKIAAELGA |
| HV7 | see Fig. 4A | DAICIALDESATWKDMKKAMEILYKLGVKKIVVLFAYDEKLIKVAAKVLHDLGAEEAAIILAFDIDDEDEFKKQVKKAL<br>ELMKKLGVDHRIIALAMTDEEKFKKLAKIAAELGADAICIALDESATWKDMKKAMEILYKLGVKKIVVLFAYDEKLIKV<br>AAKVLHDLGAEEAAIILAFDIDDEDEFKKQVKKALELMKKLGVDHRIIALAMTDEEKFKKLAKIAAELGA |

**Table S2. Crystallographic data collection and refinement statistics**

| | OT3 |
|---|---|
| **Data collection** | |
| Beamline | SSRL BL9-2 |
| Wavelength (Å) | 0.97946 |
| Space group | $P\ 2_1\ 2_1\ 2_1$ |
| Cell dimensions | |
| *a, b, c* (Å) | 44.85, 65.53, 77.69 |
| *α, β, γ* (°) | 90, 90, 90 |
| Unit cell volume (Å$^3$) | 228,296 |
| Mosaicity (°)[a] | 1.00 |
| Wilson B factor[b] | 37.4 |
| Matthews coefficient (Å$^3$/Da)[c] | 2.23 |
| Solvent content (%) | 44.8 |
| Resolution (Å)[d] | 50.09 (1.92) |
| No. of reflections/unique | 52,130/17,768 |
| $R_{merge}$[e] | 0.048 (0.663) |
| $I / \sigma I$ ratio[f] | 8.3 (1.3) |
| CC1/2 ratio | 0.999 (0.749) |
| Completeness (%)[g] | 98.5 (98.3) |
| Redundancy[h] | 2.9 (2.9) |
| | |
| **Refinement** | |
| Resolution (Å) | 38.843-2.100 |
| No. reflections/test set | 13,666/675 |
| $R_{work}$ / $R_{free}$[i] | 22.52/24.78 |
| $F_{obs}$-$F_{calc}$ correlation[j] | 0.97 |
| No. atoms | |
| Protein | 3,843 |
| Water | 30 |
| *B*-factors | |
| Protein | 62.9 |
| R.m.s. deviations | |
| Bond lengths (Å) | 0.011 |
| Bond angles (°) | 1.345 |
| Ramachandran statistics[k] | |
| Most favored regions (%) | 99.56 |
| Disallowed regions (%) | 0.44 |

[a]Degree of crystal imperfection, a higher mosaicity contributes to broader (less sharply defined) diffraction intensity profiles

[b]Overall B-factor value, an approximation to the fall-off of atomic scattering with resolution

[c]Ratio of the volume of the asymmetric unit to the molecular weight of all protein molecules in the asymmetric unit

[d]Value in parentheses is for the highest-resolution shell: 1.92 – 2.02 Å.

[e]Reliability factor for symmetry-related reflections calculated as: $R_{merge} = \Sigma_{hkl}\Sigma j=1$ to N | $I_{hkl} - I_{hkl}$ (j) | / $\Sigma_{hkl}\Sigma j=1$ to N $I_{hkl}$ (j), where N is the redundancy of the data. In parentheses, the cumulative value at the highest-resolution shell
[f]Ratio of mean intensity to the mean standard deviation of the intensity over the entire resolution range
[g]Fraction of measured reflections to possible observations at the resolution range
[h]Number of measurements of individual, symmetry unique reflections
[i]Average deviation between the observed and calculated structure factors calculated as: $R_{work} = \Sigma_{hkl} ||F_{obs}| - |F_{calc}|| / \Sigma_{hkl} |F_{obs}|$, where the $F_{obs}$ and $F_{calc}$ are the observed and calculated structure factor amplitudes of reflection hkl. $R_{free}$ is equal to $R_{factor}$ but for a randomly selected 5.0 % subset of reflections that were held aside throughout refinement for cross-validation
[j]Correlation coefficient between observed and calculated structure factor amplitudes
[k]Ramachandran statistics for non-proline and non-glycine residues
